# Two sides of the same coin: heat shock proteins as biomarkers and therapeutic targets for some complex diseases 

**DOI:** 10.3389/fmolb.2025.1491227

**Published:** 2025-02-20

**Authors:** Xolani Henry Makhoba

**Affiliations:** Department of Life and Consumer Sciences, College of Agriculture and Environmental Sciences, University of South Africa (UNISA), Florida Campus, Roodepoort, South Africa

**Keywords:** heat shock proteins, biomarkers, therapeutic targets, drug targets, critical diseases

## Abstract

Heat shock proteins are molecular chaperones that play crucial roles in the folding and unfolding of complex polypeptides within the cellular system. These molecules are involved in various processes, including vesicular transport, prevention of protein aggregation in the cytosol, and cell signaling. They are also linked to autoimmunity, infection immunity, and tumor immunology. Stressors like heat shock, exposure to heavy metals, cytokines, reactive oxygen species, inflammation, and viruses can influence the production of these molecules. In complex diseases such as cancer, malaria, and COVID-19, heat shock proteins are considered both biomarkers and drug targets. The upregulation of small heat shock proteins like hsp27 and major heat shock proteins 70/90 has been recognized as crucial biomarkers and therapeutic targets for cancer. Additionally, it has been reported that the invasion of *Plasmodium falciparum*, the causative agent of malaria, leads to the upregulation of heat shock proteins such as hsp40, hsp70, and hsp90. This sudden increase is a protective mechanism from the human host and enhances the parasite’s growth, making these proteins significant as biomarkers and malarial drug targets. The presence of the SARS-CoV-2 virus in the human cellular system correlates with a substantial increase in heat shock protein 70 production from host cells. Furthermore, our research group has demonstrated that SARS-CoV-2 hijacks the host’s heat shock proteins, and we are currently developing tools to prevent the virus from utilizing the host’s protein folding system. This review aims to highlight the role of heat shock proteins as biomarkers and therapeutic targets for selected refractory diseases, focusing on cancer, malaria, and COVID-19. A fundamental molecular docking study was performed to investigate the interaction between a non-structural complex from SARS-CoV-2 and chosen small molecules, which is emphasized in this review.

## 1 Introduction

The Italian researcher Ferruccio Ritossa discovered heat shock proteins in the early 1960s while studying nucleic acid synthesis in the puffs of *Drosophila* salivary glands. The interesting part about these molecules is that his work was rejected by high-impact journals, citing that his work lacked biological importance, the most popular phrase in research especially in heat shock-related fields today (Ritossa, 1962). We value the significant contributions made by the Experientia journal in 1962, as they opened numerous research opportunities for innovative drug development to treat complex diseases such as cancer, malaria, and viral infections (De Maio et al., 2012). It is quite fascinating that the research on heat shock proteins was initially met with skepticism. However, thanks to pioneering scientists like Susan Lindquist, who took significant risks early in their careers, we now understand that heat shock proteins respond to various stressors as a protective mechanism. Heat shock proteins play an important role in protein folding and protein homeostasis. In 2009, Kampinga and co-workers organized these vital molecules into various named families and linked to different healthy and unhealthy pathological conditions. Today, we recognize that the heat shock response is a universal mechanism triggered by various stresses, with heat shock proteins playing a crucial role in this process (Kampinga et al., 2009). These proteins are observed in numerous cellular and molecular studies, leading to an increasing number of new implications for these polypeptides. The understanding of heat shock proteins has led to several discoveries, for example, these proteins are regarded as both drug targets and biomarkers. Heat shock proteins can be used as biomarkers for cancer diagnosis and prognosis. Albakova and colleagues in 2021, studied the heat shock protein network coupled with a machine learning approach to define patients with cancer and HSP-based biomarkers of cancer (Albakova et al., 2021). The overexpression of heat shock proteins in various types of cancer cells suggested that these proteins are involved in tumor development and progression. These molecules were found in urine samples and therefore used to identify cancer patients with almost 90% precision. However, as heat shock proteins are always expressed in high levels in cancers and play a crucial role in tumor progression, their overexpression is also a prospective target for cancer therapeutics. This overexpression of these molecular chaperones can lead to drug resistance and poor prognosis.

This review will cover several interesting diseases, including malaria and COVID-19. Malaria is one of the leading causes of death in Africa, particularly in Sub-Saharan Africa, with over 249 million cases and 608,000 deaths reported globally (World malaria report). The primary causative agent of malaria is *P. falciparum,* which disproportionately affects children under the age of 5 and pregnant women, making them the most vulnerable populations. Current drugs available on the market are not effective in treating malaria. Therefore, innovative treatments are actively being researched. The survival of the malarial parasite, *P. falciparum*, has garnered significant interest in the quest for new drug development (Sesethu et al.,2023a; Vierling 1991; World malaria report, 2023). This is because the parasite can survive both in its mosquito vector and the human host, as it is transmitted through mosquito bites. When the parasite enters the human host, it produces a higher concentration of heat shock proteins as a protective response to the sudden change in temperature (Makhoba, 2021). Conversely, in the human host, the production of heat shock proteins indicates the presence of a foreign substance—in this case, the malarial parasite. Hence, these proteins are considered both drug targets and biomarkers.

The recent outbreak of coronavirus disease has claimed more than 7 million lives, with over 704,753,890 cases reported to date. This disease is caused by the SARS-CoV-2 virus, which spreads from person to person through respiratory droplets (CDC Preliminary Estimates of COVID). An interesting aspect of viruses is that they cannot produce their proteins; thus, they require a host—in this case, humans. As a result, SARS-CoV-2 uses the human host to grow, differentiate, and proliferate. SARS-CoV-2 hijacks the heat shock proteins of its human host to aid in the folding of its proteome (Makhoba and Makumire, 2022). In response, the human body increases the production of heat shock proteins as a defense against the virus. This substantial rise in heat shock proteins has been recognized as a biomarker or indicator of the presence of foreign substances, such as viruses. This review will thus, highlight the dual role of heat shock proteins as biomarkers and therapeutic targets for complex diseases such as cancer, malaria, and COVID-19. Later in the document, this review will use basic molecular docking to establish the interaction between human heat shock proteins and SARS-CoV-2 non-structural complex 7/8 (NSP7/8) with selected compounds.

## 2 A short background, nomenclature, and classification of heat shock proteins

Heat shock proteins (HSPs) can be found in plant and animal cells. They were first discovered when an Italian scientist accidentally increased the incubation temperature of *Drosophila melanogaster*, causing a significant increase in a group of proteins later identified as heat shock proteins (Ritossa, 1962). Despite being originally described as being induced by heat (Ritossa, 1962), it is now known that hsps can be induced by a wide variety of stressors, including exposure to cold, UV light, wound healing, tissue remodeling, and biotic stresses (Ritossa, 1962; Kampinga et al., 2009). Many decades later, Kampinga and his colleague proposed comprehensive guidelines for naming the human HSP families. They suggested designating the heat shock proteins from mammalian cells as, hspC (hsp90), hspA (hsp70), DNAJ (hsp40), and HSPB (small hsp), as well as for the human chaperonin families HSPD/E (hsp60/hsp10) and CCT (TRiC). This uniform naming system was adopted in this review due to the predominant focus on human heat shock proteins (Kampinga et al., 2009). Heat shock proteins vary in size and are classified by weight into small heat shock proteins (hspB), middle heat shock proteins (DnaJ), and major heat shock proteins (hspA, hspC, and hspH). Some of these heat shock proteins serve as holdases, while others function as foldases (see Table 1). Most of these proteins exhibit cooperative behavior, with some acting as co-chaperones and others as primary chaperones in their functional activities.

**TABLE 1:** Summary of HSPs in mammalian cells.

Gene name	Protein name	Alternative names
HSPC (HSP90) family		
1 hspC1^a^	HSPC1	HSP90AA1; HSPN; LAP2; HSP86; HSPC1; HSPCA; HSP89; HSP90 HSP90A; HSP90N; HSPCAL1; HSPCAL4; FLJ31884
2 hspC2^a^	HSPC2	HSP90AA2; HSPCA; HSPCAL3; HSP90ALPHA
3 hspC3^a^	HSPC3	HSP90AB1; HSPC2; HSPCB; D6S182; HSP90B; FLJ26984; HSP90-BETA
4 hspC4^a^	HSPC4	HSP90B1; ECGP; GP96; TRA1; GRP94; endoplasmin
5 hspC5^a^	HSPC5	TRAP1; HSP75; HSP90L
HSPA (HSP70) family		
1 hspA1A	HSPA1A	HSP70-1; HSP72; HSPA1
2 hspA1B	HSPA1B	HSP70-2
3 hspA1L	HSPA1L	hum70t; hum70t; Hsp-hom
4 hspA2	HSPA2	Heat-shock 70kD protein-2
5 hspA5	HSPA5	BIP; GRP78; MIF2
6 hspA6	HSPA6	Heat shock 70kD protein 6 (HSP70B′)
7 hspA7^a^	HSPA7	Heat shock 70kD protein 7
8 hspA8	HSPA8	HSC70; HSC71; HSP71; HSP73
9 hspA9	HSPA9	GRP75; HSPA9B; MOT; MOT2; PBP74; mot-2
10 hspA12A	HSPA12A	FLJ13874; KIAA0417
11 hspA12B	HSPA12B	RP23-32L15.1; 2700081N06Rik
12 hspA13^b^	HSPA13	Stch
13 hspA14	HSPA14	HSP70-4; HSP70L1; MGC131990
HSPH		
1 hspH1	HSPH1	HSP105
2 hspH2^b^	HSPH2	HSPA4; APG-2; HSP110
3 hspH3^b^	HSPH3	HSPA4L; APG-1
4 hspH4^b^	HSPH4	HYOU1/Grp170; ORP150; HSP12A
DnaJA (HSP40) family		
1 DnaJA1	DnaJA1	DJ-2; DjA1; HDJ2; HSDJ; HSJ2; HSPF4; hDJ-2
2 DnaJA2	DnaJA2	DNJ3; mDj3; Dnaj3; HIRIP4
3 DnaJA3	DnaJA3	Tid-1; Tid1l
4 DnaJA4	DnaJA4	Dj4; Hsj4
DnaJB		
5 DnaJB1	DnaJB1	HSPF1; HSP40
6 DnaJB2	DnaJB2	HSJ1; HSPF3; Dnajb10; MDJ8
7 DnaJB3	DnaJB3	Hsj3; Msj1; MSJ-1; Hcg3a
8 DnaJB4	DnaJB4	Hsc40
9 DnaJB5	DnaJB5	Hsc40; HSP40-3
10 DnaJB6	DnaJB6	Mrj; mDj4
11 DnaJB7	DnaJB7	Dj5; mDj5
12 DnaJB8	DnaJB8	mDj6
13 DnaJB9	DnaJB9	Mdg1; mDj7; ERdj4
14 DnaJB11	DnaJB11	Dj9; ABBP-2; Erdj3
15 DnaJB12	DnaJB12	Dj10; mDj10
16 DnaJB13	DnaJB13	Tsarg6; Tsarg 3 protein
17 DnaJB14	DnaJB14	EGNR9427; FLJ14281
DNAJC		
19 DanJC1	DanJC1	MTJ1; ERdj1; ERj1p; Dnajl1
20 DnaJC2^b^	DanJC2	Zrf1; Zrf2; MIDA1; M-phase phosphatase protein 11 MPP11; zuotin; ZUO1
21 DnaJC3	DanJC3	p58; mp58; Prkri; Dnajc3; p58IPK; Dnajc3b
22 DnaJC4	DanJC4	HSPf2; Mcg18
23 DnaJC5	DanJC5	Csp
24 DnaJC5B	DanJC5B	Csp-beta
25 DnaJC5G	DanJC5G	MGC107182; gamma-CSP
26 DnaJC6	DanJC6	mKIAA0473; auxilin
27 DnaJC7	DanJC7	Ttc2; mDj11; mTpr2
28 DnaJC8	DanJC8	AL024084; AU019262; splicing protein (spf31)
29 DnaJC9	DanJC9	AU020082; RcDNAJ9
30 DnaJC 10	DanJC10	JPDI; ERdj5; macrothioredoxin
31 DnaJC11	DanJC11	FLJ10737; dJ126A5.1
32 DnaJC12	DanJC12	Jdp1; mJDP1
33 DnaJC13	DanJC13	Rme8; RME-8; Gm1124
34 DnaJC14	DanJC14	HDJ3; LIP6; DRIP78
35 DnaJC15	DanJC15	Dnajd1; MCJ; Cell growth-inhibiting 22 protein
36 DnaJC16	DanJC16	mKIAA0962
37 DnaJC17	DanJC17	C87112
38 DnaJC18	DanJC18	MGC29463
39 DnaJC19	DanJC19	TIM14; TIMM14
40 DnaJC20^b^	DanJC20	JAC1; HSC20; HscB
41 DnaJC21	DanJC21	GS3; JJJ1; DNAJA5
42 DnaJC22	DanJC22	FLJ13236; Wurst
43 DnaJC23^b^	DanJC23	Sec63; AI649014
44 DnaJC24^b^	DanJC24	DPH4; zinc finger, CSL-type containing 3
45 DnaJC25	DanJC25	bA16L21.2.1; DnaJ-like protein; AAH48318; LOC552891 G-protein gamma 10
46 DnaJC26	DanJC26	GAK; cyclin G associated kinase; auxilin-2
47 DnaJC27^b^	DanJC27	RBJ; RabJ
48 DnaJC28	DanJC28	Orf28 open reading frame 28; C21orf55, oculomedin
49 DnaJC29^b^		Sacsin; SACS
50 DnaJC30	DanJC30	WBSCR18; Williams–Beuren syndrome chromosome region 18 homolog (human)
HSPB (HSP27) small heat shock protein family		
1 hspB1	HSPB1	CMT2F; HMN2B; HSP27; HSP28; HSP25; HS.76067; DKFZp586P1322
2 hspB2	HSPB2	MKBP; HSP27; Hs.78846; LOH11CR1K; MGC133245
3 hspB3	HSPB3	HSPL27
4 hspB4^a^	HSPB4	crystallin alpha A; CRYAA, CRYA1
5 hspB5^a^	HSPB5	crystallin alpha B, CRYAB; CRYA2
6 hspB6	HSPB6	HSP20; FLJ32389
7 hspB7	HSPB7	cvHSP; FLJ32733; DKFZp779D0968
8 hspB8	HSPB8	H11; HMN2; CMT2L; DHMN2; E2IG1; HMN2A; HSP22
9 hspB9	HSPB9	FLJ27437
10 hspB10^a^	HSPB10	ODF1; ODF; RT7; ODF2; ODFP; SODF; ODF27; ODFPG ODFPGA; ODFPGB; MGC129928; MGC129929
11 hspB11	HSPB11	HSP16.2; C1orf41; PP25

^a^ Annotated as a pseudogene, but possibly a true gene.

^b^ Under consultation with HGNC, and the scientific community.

## 3 Heat shock protein subtype structure

### 3.1 HSP90 family (hspC)

**FIGURE 1:**
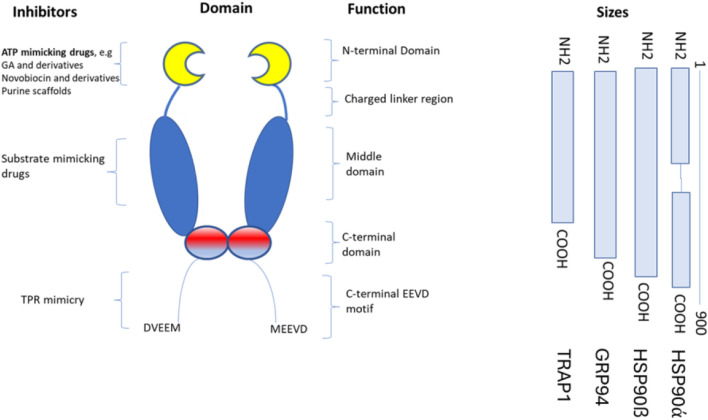
The structure of hspC and its subdomains that are involved in the binding of newly synthesized proteins. The hspC protein has different structural domains within its dimer. These include the N-terminal domain (NTD), which binds to ATP, GA, and other small molecule inhibitors and interacts with co-chaperones and potential client proteins. Following the NTD is an unstructured, highly charged linker region, which is not present in HtpG. The middle domain (MD) contains a catalytic arginine necessary for ATPase activity, binds to co-chaperones, and is believed to be the primary site for binding client proteins. The C-terminal domain (CTD) contains the main dimerization interface, which allows hspC to form a dimer structure. Finally, at the C-terminus, a highly conserved MEEVD motif binds to TPR-containing co-chaperones.

The classification of hspC proteins is based on their compartmentalization. For example, cytoplasmic proteins are grouped as hspC1 (hsp 90-alpha), hspC2 (hsp 90-alpha A2), and hspC3 (hsp 90-beta), ER-resident member; hsoC4 or GRP94 (GP96), and mitochondrial localized member; hspC5 (TRAP1) (Figure 1). Other hspC proteins are classified based on their expression pattern, where hspCβ is constitutively expressed, while hspCα is induced in stressful conditions. The structural, biochemical, and molecular characteristics of hsp90 have been extensively reviewed. The molecular structure of hspC homologs is made of domains, namely: the N-terminal domain (NTD), C-terminal domain (CTD), and middle domain (MD). In eukaryotes, a variable linker domain exists that connects the N-terminal domain (NTD) to the middle domain (MD) (Borges and Ramos, 2005). Each domain within the hspC90 structure serves a specific function. The NTD binds to ATP, hence it is referred to as the nucleotide-binding site. The dimerization of protein occurs in the CTD. The CTD consists of critical motifs such as MEEVD or KDEL which are solely on the hspC structure and regulates its localization in the cellular system be it ER or cytoplasm. Although the charged linker domain has a divergent sequence among many eukaryotic organisms, it is crucial for the chaperone function, interaction, and flexibility (Kayastha et al., 2024). HspC is responsible for numerous functions, such as the maturation, structural maintenance, and proper regulation of specific target proteins involved in cell cycle control and signal transduction. ATP plays a key role in the functional activities of HspC in the cellular system. This functional cycle could promote structural changes in the substrates, leading to their activation. It interacts dynamically with various co-chaperones that modulate its substrate recognition, ATPase cycle, and chaperone function.

### 3.2 hspC interacts with different proteins

**FIGURE 2:**
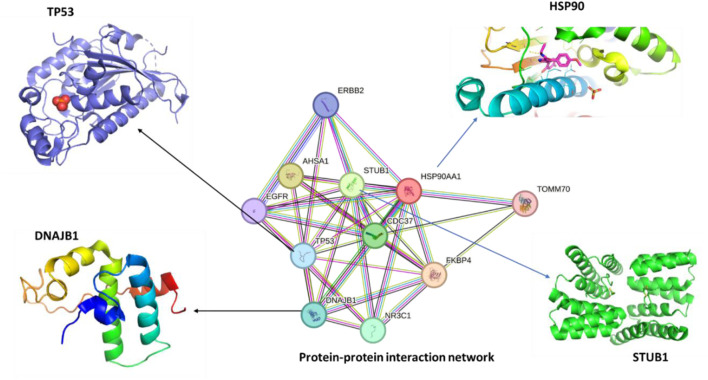
The protein-protein interaction (PPI) network of human hspC (hsp90, UniProt, ID:O14818) was obtained using STRING, and 3D structures of the most related proteins, DnaJB1 (UniProt, ID: P25685), STUB1 (UniProt, ID: Q9UNE7), and TP53 (UniProt, ID: Q12888) being the most crucial protein in cancer and other diseases.

The STRING database for known protein-protein interactions was used to identify proteins interacting with human hspC. The protein sequences of hspC and the selected interacting proteins were obtained from the protein data bank via STRING. Among the selected proteins, E3 ubiquitin-protein ligase was found to have close interactions with hspC. This ligase collaborates with ATXN3 in the degradation of misfolded chaperone substrates. ATXN3 restricts the length of the ubiquitin chain attached to STUB1/CHIP substrates and prevents further chain extension. Additionally, it ubiquitinates NOS1 in concert with hspA and DnaJC, thereby modulating the activity of several chaperone complexes, including hspA and hspC (Figure 2) (Chavez et al., 2016). Cellular tumor antigen p53, which acts as a tumor suppressor in many tumor types, was also identified. It induces growth arrest or apoptosis and is involved in cell cycle regulation as a trans-activator negatively regulating cell division. Furthermore, DnaJ homolog subfamily B member 1 was found to interact with hspA and stimulate ATPase activity. Moreover, heat shock protein hspC-alpha, a molecular chaperone, plays a vital role in promoting the maturation, structural maintenance, and proper regulation of specific target proteins involved in cell cycle control and signal transduction (Lindquist and Craig, 1988). By inducing conformational changes in client proteins and interacting with different co-chaperones to modulate its substrate recognition, ATPase cycle, and chaperone function, this chaperone goes through a functional cycle that is linked to its ATPase activity.

### 3.3 HSP70 family (hspA)

**FIGURE 3:**
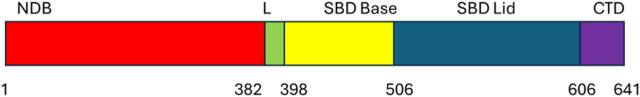
Domain organization of heat shock protein 70.

Heat shock protein 70 comprises of three important subdomains, namely: the N-terminal ATPase domain, which is 44 kDa and binds and hydrolyzes ATP to ADP, generating energy for chaperone activity (Sharma and Masison, 2009). This crucial domain contains subdomains (IA, IB, IIA, IIB) which surround the ATPase binding pocket. This domain is followed by the 28 kDa substrate binding domain (SBD), which is responsible for binding newly synthesized client proteins for proper folding (Figure 3). This domain consists of the 15 kDa beta-sheet and the 10 kDa helical subdomains. The beta-sheet subdomain has a groove that binds to hydrophobic and neutral amino acids. The C-terminal domain (CTD), which is 10 kDa, is the last domain structure found in the HSP70 structure and forms a lid-like structure over the SBD to assist in trapping the substrate.

### 3.4 HSP60 family

**FIGURE 4:**
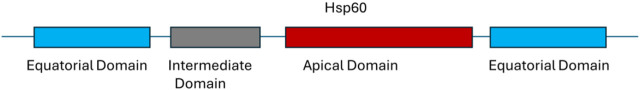
Domain organization of heat shock protein 60.

Heat shock proteins (HSPs) are highly conserved proteins present in all types of cells. Among the 10 identified families, hsp60 stands out as a crucial player. Functioning as ATP-dependent molecular chaperones in the cytoplasm, hsp60 aids in the folding of newly synthesized polypeptides and the assemblingy of multiprotein complexes (Figure 4). Notably, hsp60 is expressed on the cell surface, particularly in bacteria, where its expression increases significantly during host infection and in biofilm formation (MaleKi et al., 2016). In the extracellular environment, hsp60 can act as a crucial link between immune cells, impacting the immune system’s activity in various ways. Furthermore, HSPs have demonstrated their potential as potent activators of the immune system and are considered strong candidates for subunit vaccines or adjuvants (Hong et al., 2024).

### 3.5 HSP40 family (dnaJ)

**FIGURE 5:**
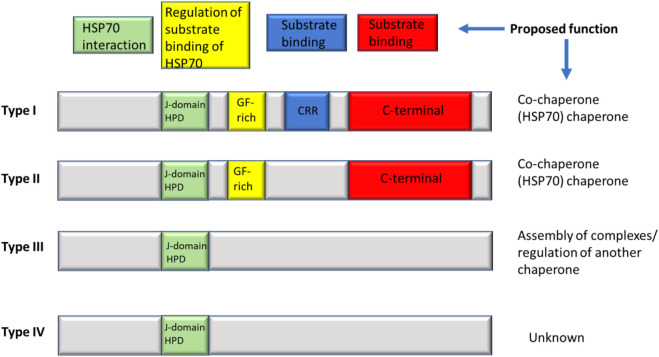
The classification of J-proteins includes four types. Type I comprises 4 domains, with the J-domain found on the N-terminal end with a G-F rich region, the Zinc binding domain follows which ends in the substrate binding site of the C-terminal domain. While TypeII shares the J-domain and G-F-rich region it does not consist of the zinc-finger region. Type III protein only has a J-domain, while Type IV only lacks the most conserved HPD motif; zinc-regions are also unavailable in these types of proteins.

The consistent synthesis of polypeptides within the cellular system is crucial for proper cellular function. However, this process occurs in a complex and crowded environment. Molecular chaperones, particularly heat shock proteins (hsps), play a pivotal role in ensuring the quality and functionality of newly synthesized proteins (Qiu et al., 2006). HspA is a key player in maintaining protein homeostasis. Its adaptability and specificity in substrate binding depend significantly on its interaction with co-chaperones such as DnaJCs, JDPs, and J-proteins (Figure 5). These interactions are essential for coordinating HspA’s two major domains, the substrate binding domain (SBD) and the nucleotide-binding domain (NBD) (Qiu et al., 2006). The transient interaction of the SBD with target proteins is modulated by an allosteric mechanism involving ATP hydrolysis and ADP/ATP exchange in the NBD, highlighting the sophisticated regulatory processes involved in protein synthesis and function.

The JDPs have a dual role: they help substrate proteins bind to hspA in its ATP-bound state. At the same time, a direct interaction between a JDP and hspA triggers the hydrolysis of ATP and causes hspA to transition to its ADP-bound state, which has a high affinity for the specific polypeptide brought over by a particular JDP. Another important factor is the nucleotide exchange factor (NEF), which promotes the dissociation of ADP and the binding of a new ATP molecule. This modification changes the hspA structure to a low-affinity state, leading to the release of the substrate (Qiu et al., 2006; Boston et al., 1996).

### 3.6 DnaJ/hspA (hsp40/hsp70) cooperate with hspC (hsp90) in chaperone activities

**FIGURE 6:**
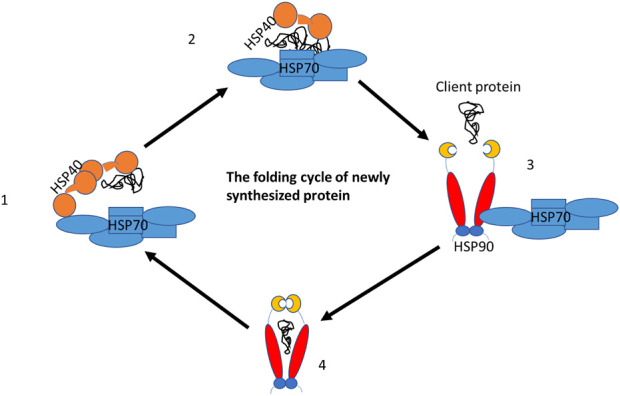
The folding cycle of the newly synthesized proteins with the help of chaperone partners. DnajC (Hsp40) cochaperone binds to the new proteins and hands it over to the hspA (Hsp70) chaperone for folding. However, in case the protein needs further folding, Hsp70 hands it over to hspC (Hsp90) for the completion of folding and releases it as a fully functional protein.

Molecular chaperones cooperate in the folding process of the newly synthesized polypeptide. For example, dnaJC binds the client proteins as a cochaperone and hands them over to hspA to assist newly synthesized proteins in completing their folding to a three-dimensional structure. This structure is fully functional, however, if the protein has not fully achieved its functional activities it is handed over to hspC for further folding (Figure 6). The hspA and hspC molecular chaperone systems are essential for regulating protein homeostasis in eukaryotic cells under normal and stressful conditions. Both hspA and hspC work together to maintain proteostasis in the cell. Their functions are activated by cochaperones, which interact with either hspA or hspC (Olivier et al., 2019). The human heat shock organizing protein (Hop) and its yeast ortholog Stip1 are eukaryotic cochaperones that play a critical role in transferring client proteins from hspA to hspC. This transfer is regulated by Hop, as it can interact with both hspA and hspC simultaneously, forming a ternary complex. Unlike eukaryotes, prokaryotes do not have a Hop ortholog, so the hspA and hspC directly interact. It is believed that eukaryotic hspA and hspC can also form a prokaryote-like binary chaperone complex in the absence of Hop (Pedrola and Rüdiger, 2021). This binary complex exhibits enhanced protein folding and anti-aggregation activities.

### 3.7 Small heat shock proteins (hspB)

**FIGURE 7:**
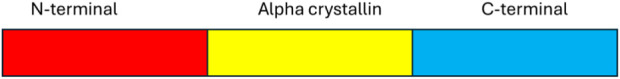
Small heat shock protein domain structure.

Small heat shock proteins are low molecular weight proteins ranging between 12 and 43 kDa. There are various types of sHSPs, which include HSPB1-8. Alpha crystallin 6 is the most studied family due to its major presence in the human eye lens (Figure 7). sHSPs form large oligomeric complexes under physiological conditions. The rate of disassembly influences their chaperoning activities. Both heat and other environmental conditions contribute to changes in their structure (Tedesco et al., 2022). The main role of small heat shock proteins is to bind to partially denatured polypeptides to prevent irreversible aggregation under stressful conditions. Small heat shock proteins form part of the cellular protein quality control system. The figure below shows the crucial domains that constitute the small heat shock proteins, with the alpha-crystallin domain (ACD) in the middle, followed by a flexible N-terminal region (NTR) at one end and a short C-terminal region (CTR) on the other side (Tedesco et al., 2022). About 90 amino acids are found in the ACD, a conserved domain made up of beta strands that form dimers, the basic building blocks of sHSPs. Both NTR and CTR play crucial roles in oligomerization.

## 4 Physiological function

Heat shock proteins are molecular chaperones found in bacteria and humans. These molecules vary in size, from small to major heat shock proteins. Their role is to assist other proteins in folding properly, and their functional activities are generally cooperative. For example, hsp40 identifies client proteins and activates the activity of hsp70, thus handing over the client protein for folding processes. If the client protein needs further folding, hsp70 transfers it to hsp90 for proper folding (Bhattacharya et al., 2020). Table 2 overviews the heat shock proteins and their functional activities. Two functional activities stand out. Foldases, for instance, bind to newly produced proteins to help them fold correctly with the use of ATP. In contrast, holdases bind to newly synthesized proteins to prevent the formation of aggregates but do not utilize ATP.

**TABLE 2:** A general view of heat shock proteins and their functional activities.

General name	Molecular mass	Function	*E. coli* gene	Yeat gene	Human gene
Hsp100	∼100/hexamer	Holdase/diaggregase	clipB	hsp104	clpx
Hsp90	∼90/dimer	Foldase	htpG	hsp82	hps90
Hsp70	∼70/monomer	Foldase	dnaK	Ssa, ssb	hspa
Hsp60/10	∼60/∼10 double heptamer/heptamer	Foldase	groEL/groES	Hsp60/hsp10	Hspd/hspa
Hsp40	∼40/dimer	Holdase	dnaJ	Ydj1 sis1	dja1, dja2, dja4
Small Hsp	∼10-30/8-24	Holdase	ibpA, ibpB	sm Hsp	crya, hspB

## 5 Heat shock proteins as disease biomarkers

Heat shock proteins (HSPs) are being explored as biomarkers for various diseases. Their overproduction during stress makes them valuable for diagnosing and treating conditions such as cancer, malaria, and COVID-19. Specifically, hsp27, hsp40, hsp70, and hsp90 are frequently overexpressed in cancer cells, aiding their survival in stressful environments. This positions them as potential biomarkers for cancer detection and prognosis. Current research focuses on understanding the roles of HSPs in *P. falciparum* and their utility as biomarkers, including how they assist the parasite in coping with stress and identifying unique motifs suitable for therapeutic targeting. Hsp27 has been recognized as crucial in the development of COVID-19, especially in severe cases leading to acute respiratory distress syndrome (ARDS). Higher blood levels of hsp27 correlate with increased systemic inflammation and poorer clinical outcomes in COVID-19 patients. Table 3 gives the names, roles, and tissues where heat shock proteins are mostly expressed (Caillet et al., 2022; Navhaya et al., 2024).

**TABLE 3:** Summary of heat shock proteins as ideal biomarkers for different diseases.

HSP Isomers	Roles in cancer, malaria and COVID-19	Tissue
Hsp90 (HSP90α, HSP90β, GRP94 and TRAP1)-Cancer PfHSP90, GRP94-Malaria HSP90α, HSP90β, GRP94 and TRAP1-COVID-19	Elevated hsp90 expression is associated with the progression of cancer, the survival of malarial parasites, and viral activity replication	Lung cancer, esophageal carcinoma, bladder cancers, lever cells, and erythrocytes. Red blood cells
Hsp70 (HSPA1A, HSPA8, HSPA5, and HSPA1AB)-Cancer PfHSP70-1, PfHSP70-x, GRP78 (PfHSP70-2)-Malaria HSPA1A, HSPA5-COVID-19	Increased expression of hsp70 contributes to tumor cell progression, and its release into the extracellular space makes it an ideal biomarker to monitor the outcome of radiation therapy and other cancer treatments. Malarial parasites use this chaperone protein to survive sudden changes in temperature. Viruses use hsp70 for protein folding, thereby hijacking the human host’s chaperone system	Lung cells, red blood cells, liver cells
Hsp40 (DanJB1, DanJA1, DnaJB6)-Cancer PfHSP40 and PfHP40-x-Malaria DnaJB1 and DnaJA1-COVID-19	Heat shock protein overexpression is reported in many cancers such as gastric, colorectal, cervical, and lung cancers. This crucial chaperone is linked to cancer cell proliferation, metastasis, and invasion. In the malarial parasite, this molecule is known for its role as a co-chaperone, thus identifying and bringing client proteins to hsp70 and hsp90 for folding. It also facilitates the viral protein synthesis and folding	Lungs, red blood cells, liver cells
HSPB (small heat shock proteins) (HspB1, HSPB5 (αB-crystallin), HspB8-Cancer HspB1 and HspB5 (αB-crystallin)-Malaria HspB1-COVID1-19	Small heat shock proteins are found in higher levels in various cancers, such as breast, lung, and gastric cancers. They play crucial roles in the advancement of cancer and could serve as promising targets for treatment	Lungs, gastric, and urine, and red blood cells, kidney cells

## 6 Heat shock proteins as drug targets

Heat shock proteins (HSPs) are becoming prime drug targets for a range of diseases, including cancer, malaria, and COVID-19. Particularly, hsp90, hsp70, and hsp27 are frequently overproduced in cancer cells, which aids their survival and growth. By targeting these proteins, multiple critical signaling pathways necessary for tumor growth and persistence can be interrupted. In the case of *P. falciparum*, the malaria-causing parasite, HSPs are vital for its life cycle. They enable the parasite to withstand the challenging conditions in both the human host and mosquito vector. Additionally, HSPs participate in the inflammatory and cellular stress responses linked to COVID-19 and are currently being investigated as both biomarkers and therapeutic targets (Navhaya et al., 2024; Lee et al., 2015). The concentration ranges of heat shock proteins vary significantly under physiological and pathological conditions, as highlighted in Table 4.

**TABLE 4:** Concentration ranges of HSPs under physiological and pathological conditions.

General name of HSP	Concentration under physiological conditions	Concentration under pathological conditions
Hsp90	1–10 ng/mL serum	At the disease state 50–200 ng/mL
Hsp70	0.1–1 ng/mL in serum	At the disease state, 10–100 ng/mL
Hsp40	0.1–1 ng/mL serum	5–20 ng/mL
Small heat shock proteins	0.1–1 ng/mL serum	10–50 ng/mL

### 6.1 Advanced techniques for detecting and measuring heat shock proteins

Multiple techniques exist to detect and measure the expression patterns of heat shock proteins in both normal and pathological conditions. Mass spectrometry (MS) is crucial for identifying and quantifying these proteins, offering insights into their structural and chemical properties, which is essential for proteomics research and biomarker discovery. In cell and tissue extracts, the enzyme-linked immunosorbent assay (ELISA) is another prominent method used to quantify heat shock proteins, including HSP90α and HSP27 (Lee et al., 2015). This highly sensitive technique can identify low protein levels. The Western blot method is also valuable for protein detection and measurement; it involves gel electrophoresis of proteins followed by transferring them to a membrane to detect specific heat shock proteins using antibodies. This method yields information on the molecular weight and expression patterns of these proteins. Immunohistochemistry is a technique applied to detect proteins in tissue samples by staining them with specific antibodies targeting heat shock proteins, enabling visualization of protein localization and expression within tissues. Lastly, bead-based immunoassays provide high sensitivity and specificity, allowing for multiplexing to detect multiple heat shock proteins simultaneously (Xia, 2017; Njemini et al., 2008).

#### 6.1.1 Heat shock proteins as drug targets for cancer

**FIGURE 8:**
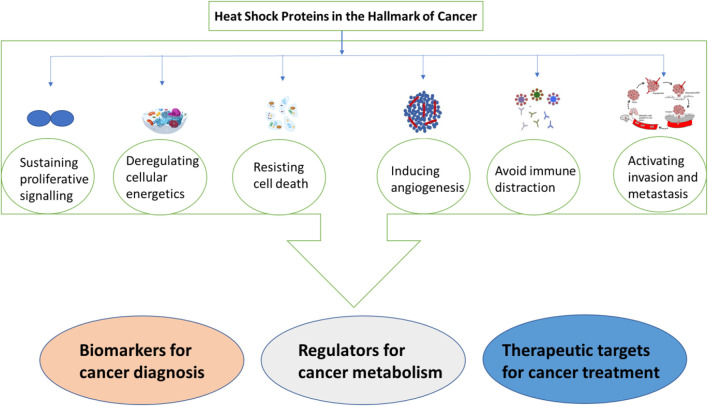
A summary of heat shock proteins as biomarkers for cancer.

Heat shock proteins (HSPs) are involved in cancer development, resistance to apoptosis, invasion, and angiogenesis. They also promote immune tolerance and proliferation. HSPs are thought to be possible biomarkers for cancer because of a study that revealed an increase in HSPs in malignant cells, such as in liquid biopsies of cancer patients. Increased expression of hsps has also been seen in other cancers, including lung cancer, and is typically linked to tumor cell survival, invasion, metastasis, and resistance to chemotherapy (Welch, 1993). In addition to that hsp90 was reported to show a sensitivity of 85% and specificity of 90% in detecting early stages of breast cancer. Therefore, high production of hsp90 has been associated with tumor aggressiveness and resistance to chemotherapy (Welch, 1993). Hsps interact with various oncogenes to propel cancer progression. Additionally, cells with elevated expression of hsps have been shielded against radiation-induced cell death. This suggests that cancer development, proliferation, and growth rely on increased expression of hsps, especially under stressful conditions. The transcriptomic and proteomic studies conducted on the lung tumor expression profile depicted a correlation between HSP expression levels. This is regarded as a potential marker for cancer and could lead to better management of patients with cancer (Welch, 1993). The poor prognosis associated with elevated hsp levels in lung cancer patients prompts the consideration of these proteins as therapeutic targets in addition to the existing lung cancer treatment strategies. Figure 8 summarizes the role played by hsps as both therapeutic targets and biomarkers in cancer. It is reported that samples (plasma, serum, and plasma/urine-derived exosomes) collected from patients with cancer showed high expression levels of various hsps, such as hspA, hspC, DnaJ, and hspB, compared to those from healthy participants (Gunaldi et al., 2015; Krawczyk et al., 2020). This discovery has brought new insights into the use of HSPs as biomarkers for cancer.

Hypoxic, acidotic and nutrient-deprived circumstances can lead to high expression of heat shock proteins (hsps) in different cancers as part of the body’s response mechanism. Increased production of hsps could contribute to the formation of an immunosuppressive tumor environment, exacerbating tumor growth, hindering cancer treatment, and causing drug resistance. This can also lead to heightened expression of oncoproteins, which rely on hsps to stabilize, fold, and aggregate (Lebret et al., 2003). Therefore, it is crucial to develop tools that can regulate both the expression and functional activities of hsps to prevent their role in promoting tumor development and malignant growth. Targeting the hsps as a molecular chaperone family could be important for developing novel diagnostic and therapeutic approaches.

#### 6.1.2 HspB and its various activities in cancer

**FIGURE 9:**
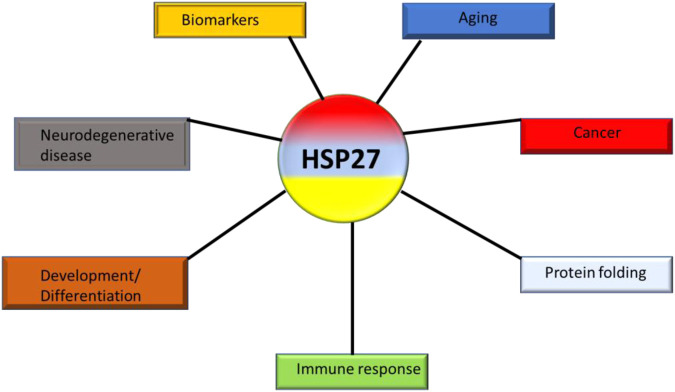
The various roles of hspB (hsp27) in cancers.

Small heat shock proteins, despite their low molecular weight, play a critical role as molecular chaperones (Figure 9). Their functional activities are controlled by phosphorylation and post-translational structural changes. Structural modifications of hspB or abnormal phosphorylation contribute to the development and proliferation of cancer (Seigneuric et al., 2011). High levels of hspB expression have been associated with the development of lung cancer and are believed to contribute to poor prognosis, chemo-resistance in NSCLC, laryngeal cancers, and lung cancer stem-like cells (Saini and Sharma, 2018; Tan et al., 2022). *In vitro* studies suggest that increased levels of hspB promote metastatic behavior and drug resistance. Additionally, high expression of hspB in tumor tissues has been found to correlate with elevated levels in serum samples of NSCLC patients compared to healthy controls. This difference is notable even in the early stages of NSCLC tumors and is related to advanced cancer development and TNM staging. The differential expression of hspB in lung tumors, including NSCLC, and its association with tumor advancement and resistance to conventional therapies suggests the potential for anti-cancer therapeutic strategies to be developed.

The recent advancements in understanding the molecular mechanisms associated with the development and progression of lung cancer have led to the discovery of several new molecular targets. Among these targets, heat shock proteins (hsps) have been identified as potential targets for anticancer treatments due to their involvement in the progression of lung tumors. Because hsps play a regulatory role in both normal and pathological conditions, two approaches are being considered to target hsps: one is to regulate their expression levels and activity, and the other is to develop HSP-based immunotherapies. In a study conducted by Albakova and others in 2021, it was found that heat shock proteins are expressed differently in various cancer cells. The study analyzed urine samples from cancer patients and healthy individuals []. The research showed that the levels of expression of heat shock proteins varied among different cancer types such as lung, bladder, cervical, colorectal, esophageal, and gastric cancers (Ciocca and Calderwood, 2005). This suggests that specific classes of heat shock proteins can serve as biomarkers for different types of cancer (Table 5). Tan and colleagues (2022) examined the expression levels of hspB8 in bladder cancer cells compared to healthy patients (Anas et al., 2019a). They concluded that its expression in cancer cells was higher than in healthy patients, suggesting that heat shock proteins can be used as biomarkers for cancer.

**TABLE 5:** Summary of drugs targeting HSP90, HSP70, HSP40 and HSPB in cancer.

Name of the drugs	Target	Function
1. Geldanamycin derivatives like 17-AAG, 17-DMAG	Hsp90	1. Inhibit the ATP-binding site of hsp90, thus blocking its functional activities, leading to the degradation of client proteins
2. NVP-AUY922		2. Also inhibits the ATP-binding site of hsp90 resulting in the dysfunctional activity of hsp90, thus leading to cell death
3. AT13387		3. This synthetic compound binds to hsp90 preventing its normal activity and leading to the degradation of oncogenic client proteins
4. Ganetespib (STA-900)		4. Ganetespib binds to the ATP-binding domain of hsp90 leading to the destabilizationdestabilization of multiple oncogenic proteins
1. PES (Pifithrin-μ)	Hsp70	1. Blocks the interaction between hsp70 and its client proteins, resulting in the degradation of oncogenic proteins and promoting apoptosis
2. VER-155008		2. Binds to the ATP-binding domain for hsp70 inhibiting chaperone activity leading to cancer cell death
3. MAL3-101		3. Blocking the ATPase activity of hsp70, preventing the proper folding of client proteins resulting in the apoptosis of cancer cells
4.2-Phenylethynesulfonamide (PES-Cl)		4. Binds to the substrate binding domain resulting in apoptosis
1. PES (Pifithrin-μ)	Hsp40	1. Prevent the interaction between hsp70 and hsp40, thereby disrupting their chaperone activity thus leading to the degradation of oncogenic proteins
2. VER-155008		2. Binds to the ATPase domain of hsp70, thus preventing hsp40 interaction with hsp70 thus leading to cancer cell death
3. MAL3-101		3. Also target the ATPase function of hsp70 preventing the normal function or proper folding of client proteins and promoting apoptosis in cancer cells
1. OGX-427 (Apatorsen)	Small heat shock proteins	1. Blocks the expression of hsp27 by targeting HSP27 mRNA thus directly decreasing the protein levels of hsp27 in cancer cells
2. Quercetin		2. Also prevent the production of hsp27. This compound exhibits some antioxidant properties and promotes apoptosis in cancer cells
3. RP101		3. Directly binds to hsp27, therefore preventing its functional activities, thus resulting in apoptosis in cancer cells

#### 6.1.3 Heat shock proteins as drug targets for malarial treatment

The survival of *P. falciparum* depends on two hosts, mosquito vectors and humans. This parasite is a causative agent for malaria which is a deadly disease with high cases reported in Sub-Saharan Africa. As previously mentioned, Hsps are responsible for housekeeping in the cellular system, thereby helping newly synthesized proteins to fold correctly (Alvira et al., 2014). The production of these molecules depends on sequential events, such as the activation of stress-induced heat shock protein factor-1 (HSF-1). Until recently, the exact mechanism of hsp gene activation was unclear in malarial parasites. The activation of HSF1 is also not entirely certain, but signal pathways recruited in response to heat shock have led to the activation of certain protein kinases which in turn phosphorylates HSF1. Activated HSF1 binds to 5′ promoter sequences of hsp genes and initiates transcription (Taha et al., 2019). These folding types of machinery are activated by increased or changed temperatures from mosquito vector (22°C) to human host (37°C). The human host also develops a fever, raising the temperature to 42°C, however, despite all these changes the parasite still survives. This results in a significant increase in the production of heat shock proteins as a strategy for the parasite to survive. The increased production of heat shock proteins when *P. falciparum* enters the human body is associated with the strategy used by this parasite to proliferate, grow, and develop. In addition, the increased production of heat shock proteins also signifies the presence of the parasite in the human cellular system. These proteins have attracted a lot of research attention as both biomarkers and drug targets (Andrade et al., 2022). For example, the presence of the parasite in the human host’s cellular system can be indicated by increased production of heat shock proteins (Table 6). At the same time, these proteins are perceived to be a good target for the alternative treatment of malaria. This stems from the fact that blocking their production could jeopardize the survival of the malarial parasite.

**TABLE 6:** A comparative table highlighting HSP functions as biomarkers and therapeutic targets.

HSP	Functions	Role as biomarkers	Therapeutic target
Hsp90	Cancer: Stabilizes and folds key proteins involved in cancer cell growth and survival Malaria: Facilitates folding of proteins crucial for parasite development and survival SARS-CoV-2: Facilitates folding of proteins crucial for viral replication and survival	Cancer: Overexpressed in many cancers, associated with tumor progression and resistance to therapy Malaria: Increased levels during infection, indicating a parasite stress response SARS-CoV-2: Increased levels during infection, indicating a viral stress response	Cancer: Inhibitors like geldanamycin and its derivatives disrupt cancer cell survival mechanisms Malaria: Inhibitors like geldanamycin disrupt parasite survival mechanisms SARS-CoV-2: Inhibitors like geldanamycin disrupt viral replication mechanisms
Hsp70	Cancer: Assists in protein folding, prevents aggregation, and aids in the degradation of misfolded proteins Malaria: Assists in protein folding, prevents aggregation, and aids in the degradation of misfolded proteins SARS-CoV-2: Assists in protein folding, prevents aggregation, and aids in the degradation of misfolded proteins	Cancer: Elevated levels in various cancers, indicating poor prognosis Malaria: Elevated levels during malaria infection, indicating parasite stress response SARS-CoV-2: Elevated levels in SARS-CoV-2 infection, indicating a cellular stress response	Cancer: Inhibitors like PES disrupt its function, leading to cancer cell death Malaria: Inhibitors like VER-155008 disrupt its function, impairing parasite survival SARS-CoV-2: Inhibitors like VER-155008 disrupt its function, impairing viral replication
Hsp60	Cancer: Assists in mitochondrial protein folding and protects cells from stress Malaria: Assists in mitochondrial protein folding and protects cells from stress SARS-CoV-2: Assists in mitochondrial protein folding and protects cells from stress	Cancer: Elevated levels in colorectal and gastric cancers, indicate cellular stress Malaria: Elevated levels of malaria, indicate cellular stress SARS-CoV-2: Elevated levels in SARS-CoV-2 infection, indicating cellular stress	Cancer: Targeting Hsp60 can reduce tumor growth and enhance apoptosis Malaria: Targeting Hsp60 can reduce parasite growth and enhance host immune response SARS-CoV-2: Targeting Hsp60 can reduce viral replication and enhance host immune response
Hsp40	Cancer: Co-chaperone that assists Hsp70 in protein folding and stress response Malaria: Co-chaperone that assists Hsp70 in protein folding and stress response SARS-CoV-2: Co-chaperone that assists Hsp70 in protein folding and stress response	Cancer: Increased expression in various cancers, is linked to poor outcomes Malaria: Increased expression during malaria infection, is linked to parasite survival SARS-CoV-2: Increased expression during SARS-CoV-2 infection, linked to viral survival	Cancer: Inhibitors can disrupt the Hsp70-Hsp40 complex, impairing cancer cell survival Malaria: Inhibitors can disrupt the Hsp70-Hsp40 complex, impairing parasite survival SARS-CoV-2: Inhibitors can disrupt the Hsp70-Hsp40 complex, impairing viral replication

#### 6.1.4 The hsp40 of parasite protozoa and its role in the red blood cells

**FIGURE 10:**
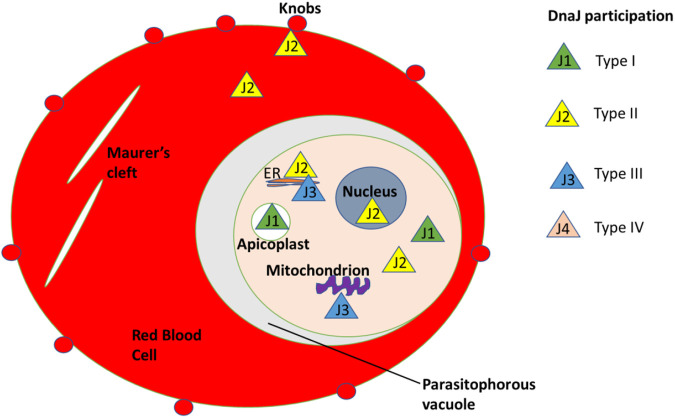
Different types of Hsp40/DnaJ compartmentalization in the red blood cells.

The heat shock protein 40 (hsp40) family in *P. falciparum* has been extensively researched to understand its role during eerythrocyte invasion. Recent studies have shown the presence of differenthsp40s in the different compartments in the red blood cells. This suggests their varied roles in protein folding, transportation, and cell signaling (Naseer et al., 2023). hsp40 proteins typically identify client proteins and facilitate the hydrolysis of ATP to ADP for protein folding by major heat shock proteins such as hsp70. The genome of *P. falciparum* contains 31 hsp40s with a signature J-domain and an additional 12 hsp40 proteins with a J-like domain where the HPD motif is modified (Figure 10). These 12 molecules have been added as a fourth class of hsp40s, previously categorized into only three classes (Type I-III). The expansion of this protein family within *P. falciparum* might be directly related to the large number of exported proteins in this malaria parasite species. Of the 43 hsp40s in *P. falciparum*, 18 contain a PEXEL motif and, of those, 5 have been experimentally proven to be exported into the host cell cytoplasm (Anas et al., 2019a).

#### 6.1.5 Molecular chaperones as malaria drug targets

Research has shown that these proteins are involved in essential processes within the parasite and the infected host cell, making them potential targets for drug development (Rubin et al., 2020). Because they aid in maintaining proteostasis during temperature changes and drug exposure, they are crucial to the parasite’s survival, virulence, and pathogenicity. These proteins are being investigated as possible targets for small molecule inhibitors because of their crucial role in essential biological functions.

## 7 Molecular docking simulation methods

### 7.1 Protein acquisition

The proteins of interest, SARS−CoV−2 nsp7/8 complex (PDB ID 7JLT), were attained from the Protein Data Bank. The molecular chaperone hsp40 was modelled using sequences obtained from UniProtKB (ID P25685), and the SWISS−MODEL tool was used to perform the homology modelling using the crystallized structure (PDB ID YDJ1) as a template. The chaperon HSP70’s 3D structure was obtained from Protein Data Bank (PDB ID 4IO8). The modelled proteins were validated using PDBsum (Negahdary et al., 2023).

### 7.2 Protein preparation

Before protein−protein docking, all three proteins were prepared using the “Protein Preparation Workflow” of the Maestro Schrodinger suite. Some of the parameters employed in the protein preparation included using a pH of 7.4 and removal of water, to mention a few. The protein nsp7/8 complex consisted of four chains (A, B, C and D). The chain names were modified to ensure the chains were treated individually and not incorporated as a single chain. That is, the chain name of hsp70 was changed to chain L, and that of hsp40 was changed to chain H (Yamkela et al., 2023).

### 7.3 Protein−protein docking

Protein-protein docking was studied to understand better the dynamics between two protein molecules. HADDOCK v2.4 (Anas et al., 2019b) was used for the docking analysis. This server was selected because it uses experimental or bioinformatic-derived data (ambiguity restraints) to drive the docking process. These data include NMR-derived intermolecular distances, residual dipolar couplings, or site-specific mutagenesis data, which help improve the accuracy of the predictions. Both proteins’ (HSP70 and HSP90) active residues were identified using information on the protein data bank and structural data (Protein Data Bank, https://www.rcsb.org/). The conformational space of the protein-protein complex was sampled by executing the docking in unbound, rigid-body, and flexible refinement phases. The HADDOCK v2.4 server was programmed to generate 1,000 dock poses; the top two hundred were chosen for further improvement. The docked complexes were analyzed using LigPlot++ (v1.4.2), a tool designed to visualize protein-ligand interactions (Olivier et al., 2019), and their binding affinities were analyzed using the AREA AFFINITY server (https://affinity.cuhk.edu.cn/index accessed on 17 May 2023). This server allows for the pasting of the docked proteins and no parameters were set on the server. Molecular dynamic simulations were conducted to evaluate the stability of the docked complexes (Sahel et al., 2024; Sesethu et al., 2023b).

### 7.4 Protein interaction analysis

The interaction analysis of the selected docking pose was performed using the “Protein Interaction Analysis” tool of BioLuminate v4.6. A two−dimensional image was generated using the specific interactions highlighted in the same Excel file for both instances. An already 2D image is available in the folder under results in the Protein−Protein docking folder.

### 7.5 Protein−ligand docking

The ligands used in this study were attained from PubChem using the following IDs: NSC11926 and NSC44175. These small molecules are naturally occurring compounds that have been used to target the SARS−CoV−2 membrane protease and the HSPA8 − Spike protein complexAutoDockTools (v1.5.7) was used in the preparation of the protein complexes (hsp40 − nsp complex and hsp70 − nsp complex. Using AutoDockTools (v1.5.7), water molecules were removed, non−polar hydrogens were merged into the protein complexes, and Kollman charges were added. The prepared structures were saved in “pdbqt” format. Before grid box generation, the SiteMap tool from Maestro as used to identify potential binding sites that were used to generate a grid box to which docking of the ligands was to occur. The following grid box information was generated: for hsp70 − nsp complex,the following coordinates were generated: npts 44; 46; 42; and centre 28.060; 20.005; 15.800 and for hsp40 − nsp complex the following were generated: npts 46; 44; 42 and centre −38.643; −1.426; 12.196. Open Babel GUI (v3.0.0) was used to convert the protonated ligands NSC11926 and NSC44175 (pH7.4 using Avogadro) from “sdf” format to “pdbqt” format in preparation for docking. AutoDockTools (v1.5.7) was used to prepare the ligand similarly to that of the protein complexes. AutoDock Vina was then used to perform protein−ligand docking, and the results were analysed using PyMOL and Ligplot^+^ (Acharya et al., 2021).

## 8. Results and discussion

### 8.1 Heat shock proteins as drug targets for COVID-19

Heat shock proteins (HSPs) are activated by various external or foreign substances such as bacteria and viruses. When SARS-CoV-2 enters the human body, the expression levels of heat shock proteins increase. For example, a study conducted by Negahdary in 2023 on COVID-19 patients using a sandwich-like system sensitive dectection for heat shock protein 70, suggested that hsp70 levels were found to be increased by approximately 50%. Therefore, making the expression of hsp70 is correlated to poor treatment of SARS-CoV-2 (Anandakrishnan et al., 2012). This increased production of heat shock proteins during a viral invasion is considered a potential biomarker. hspA8 is an important biomarker with significant potential for diagnosing and predicting the prognosis of COVID-19 (Anandakrishnan et al., 2012). Studies have shown that hspA8 exhibits varying concentration levels in COVID-19, cancer, diabetes, and certain heart diseases, making it a potential diagnostic biomarker. In a clinical study, the concentrations of hspA in the plasma of COVID-19 patients admitted to the intensive care unit (ICU) were assessed. The increased levels of hspA8 were confirmed as a biomarker predicting mortality for COVID-19 patients. This biomarker was reported to remain relatively stable at 200 ng mL−1 during a 1-week evaluation (Pattar et al., 2020; Muddagoni et al., 2021). In our laboratory, we are currently testing the role of HspA8 in its interaction with selected SARS-CoV-2 proteins. We aim to develop an alternative treatment for COVID-19. Recently, we published a comprehensive review on the interaction between the spike proteins of SARS-CoV-2 and mammalian small and major heat shock proteins. This review demonstrates how the virus hijacks the human folding machinery.

### 8.2 SARS-CoV-2 non-structural protein 7/8 complex as therapeutic targets for COVID-19

**FIGURE 11:**
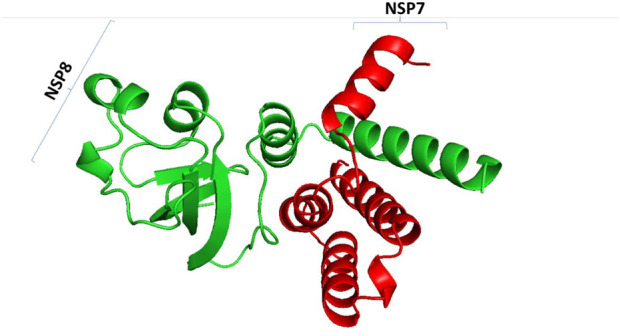
SARS-CoV-2 non-structural 7/8 complex. NSP7 is represented by red color and NSP8 is represented by blue colour.

SARS-CoV-2 has various proteins that play a crucial role in its development and replication. Spike protein and ACE-2 proteins have attracted a lot of research interest due to their role in viral entry into the human system. In addition, non-structural proteins have been identified as important molecules in the development and replication of SARS-CoV-2 (Ikwegbue et al., 2017), with more than 16 non-structural proteins from the viral genome being observed. Non-structural protein complexes have drawn much interest in the research industry, and scientists want to understand their role in the viral replication and developmental stages of the coronavirus. The SARS-CoV-2 NSP7/8 complex was sampled as proteins to understand how they interact with human host molecular folding machinery and with selected compounds to block their interaction (Figure 11).

### 8.3 HSPs on the folding of SARS-CoV-2 NSP7/8 protein complex as ideal drug targets

**FIGURE 12:**
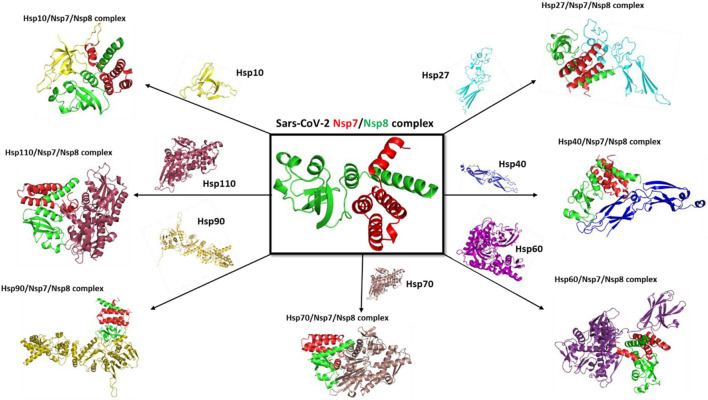
Docking complex with selected mammalian heat shock proteins with SARS-CoV-2 NSP7/8 complex.

Coronavirus replication and transcription involve multiple virus-encoded non-structural proteins (NSPs). Like other polypeptides, NSPs require assistance from molecular chaperones to form a three-dimensional structure for proper function (Figure 12). Viruses rely on human hosts to produce viral proteins by hijacking human ribosomes and heat shock proteins as folding partners. To establish the potential involvement of selected molecular chaperones in the folding stages of the viral complex protein SARS-CoV-2 NSP7/8, they were docked with the complex in a review paper. The results showed an interaction between human heat shock proteins, such as hsp10, and SARS-CoV-2 NSP7/8, indicating their role in achieving the three-dimensional structure essential for protein function. These findings suggest that viruses like SARS-CoV-2 use the human host’s folding machinery for replication (Kuramitsu, 2012). Moreover, human heat shock proteins could serve as biomarkers and potential drug targets for COVID-19 treatment. Increased levels of heat shock proteins in the presence of the virus could indicate danger within the human cellular system, while these proteins also act as molecular chaperones guiding the folding of newly produced polypeptides. This interaction between client proteins can be targeted and disrupted using drugs to interfere with the folding process.

### 8.4 The predicted binding of small molecules to heat shock proteins and NSP7/8 complex

**FIGURE 13:**
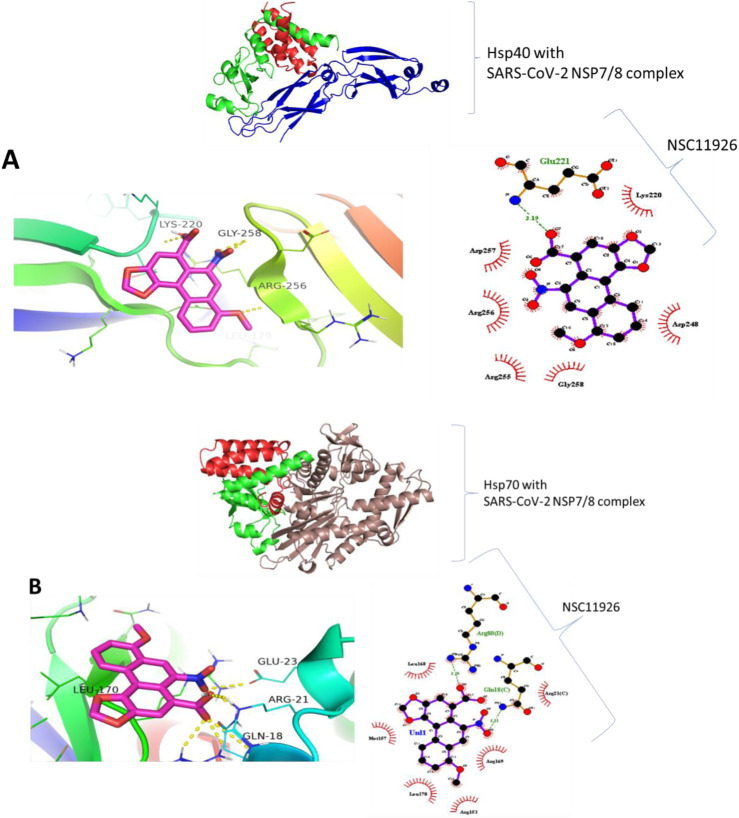
Binding prediction of 2,5-Dihydroxy-3,6-diphenyl-p-benzoquinone to **(A)** Hsp40-SARS-CoV-2 NSP7/8 complex and **(B)** Hsp70-SARS-CoV-2 NSP7/8 complex.

**FIGURE 14:**
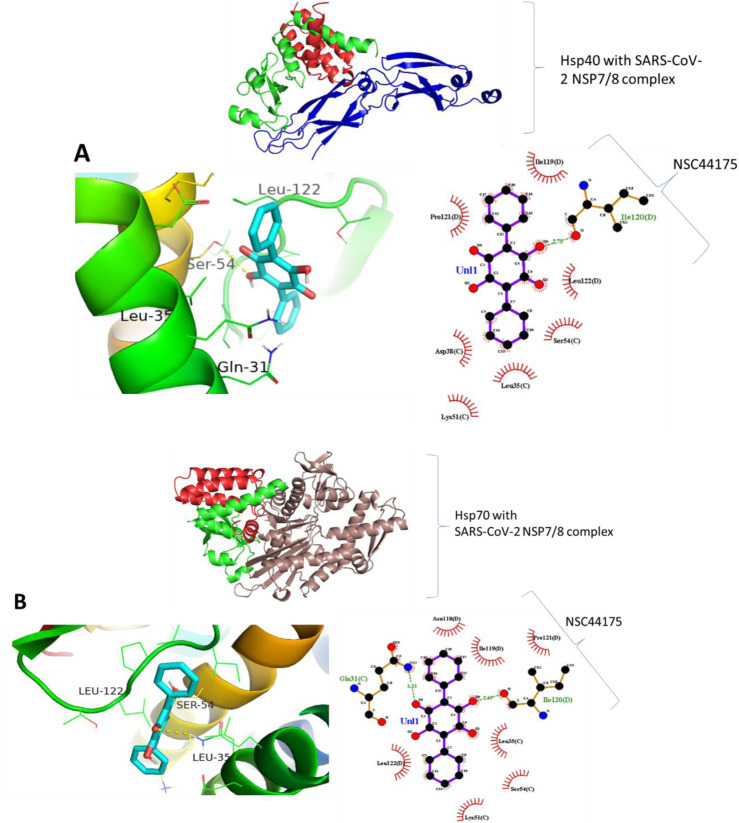
Binding prediction of Aristolochic acid to **(A)** Hsp40-SARS-CoV-2 NSP7/8 complex and **(B)** Hsp70-SARS-CoV-2 NSP7/8 complex.

Molecular docking was carried out to assess the binding of small molecules to the selected heat shock proteins (dnaJC and hspA) and SARS-CoV-2 NSP7/8 complex. Interactions were observed as shown in Figure 13 with Leucine 122, Serine 54, and Leucine 35 being the noticeable amino acids involved both in the dnaJC complex and hspA complex with 2,5-Dihydroxy-3,6-diphenyl-p-benzoquinone compound. This suggests that both folding pieces of machinery recognize this amino acid during the folding processes of the SARS-CoV-2 NSP7/8 complex. These amino acids are thus, interesting drug targets in the treatment of COVID-19. We are currently in the process of testing this treatment technique in my laboratory (Hwang et al., 2020). Also, Figure 14 shows that the hsp70 interaction was established, and certain amino acids were observed as interesting candidates in the study that is currently being conducted. It is important to note that the ligand was docked at the binding site, which was identified at the interaction location. The ligand was docked in the binding pocket of hsp70, demonstrating the most favorable binding affinity of −9.1 kcal mol−1, in comparison to the binding affinity of −6.6 kcal mol−1 (the highlighted modes) achieved when the ligand binds at the identified binding site as determined by SiteMap (Table 7). The *in vitro* study is underway in my laboratory to confirm this data in mouse models and cell culture.

**TABLE 7:** A summary of Docking scores of selected compounds with NSP7/8 SARS-CoV-2 proteins and human heat shock protein 40, and 70.

Protein	CID	Docking Score (kcal/mol)
Hsp40-nsp7/8	NSC11926	−7.2 −7 −6.9 −6.7 −6.6 −6.5
Hsp40-nsp7/8	NSC44175	−7.4 −7.4 −7 −6.9 −6.5 −6.4
Hsp70-nsp8/7	NSC11926	−7.1 −6.4 −6.3 −6.2 −6.1 −6.0
Hsp70-nsp8/7	NSC44175	−9.1 −6.6 −6.5 −6.5 −6.1 −6.1

## 9 The proposed model aims to establish molecular chaperones as biomarkers and potential drug targets

**FIGURE 15:**
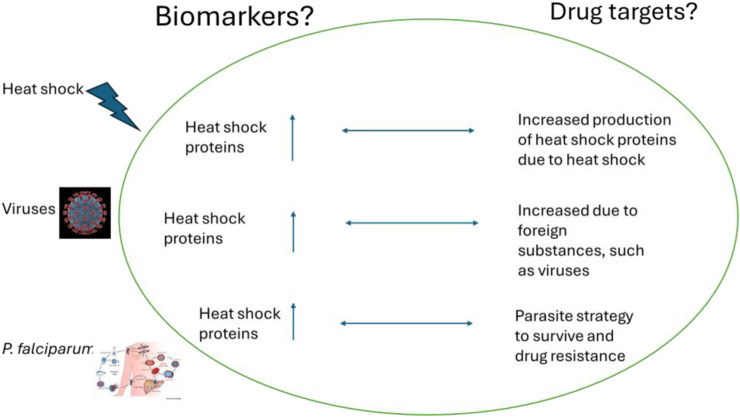
The increased production of heat shock proteins can serve as biomarkers for the presence of viruses and *P. falciparum* in the human body. Also, their presence can be ideal therapeutic drug targets.

Heat shock proteins (HSPs) are important for detecting foreign substances in the body such as viruses, parasites, and fever (Figure 15). They are also potential drug targets for treating complex diseases like malaria and cancer. In malaria, the production of molecular chaperones increases because the parasite needs them to survive. This makes them ideal drug targets since the parasite cannot survive without them. On the other hand, in the case of COVID-19, the virus cannot produce its chaperones and thus hijacks the human host chaperone system to assist in the folding processes of the viral proteins. This leads to a significant increase in chaperone proteins being produced by the mammalian system, which can serve as biomarkers, indicating the presence of the virus in the human body. For cancer, hspB and hspC are known to play a significant role in the progression of cancerous cells. Studies have shown that these molecular chaperones are found in high concentrations in cancerous cells, indicating their important role in cell proliferation and differentiation. In summary, these molecular chaperones can serve as biomarkers in cancer.

## 10 Comparative highlights of HSP functions as biomarkers and therapeutic targets across diseases

Heat shock proteins play a vital role in all living organisms and are considered important biomarkers and drug targets for various complex diseases, including cancer, malaria, and COVID-19. These proteins are categorized into different sizes and perform distinct functions within the cellular system. The table below provides a summary of their roles across different diseases (Choi et al., 2019; Owen et al., 2016).

## 11 Current limitations and challenges in HSP-based therapeutic development

Heat shock proteins (HSPs) are considered promising drug targets for various diseases. However, several limitations and challenges may hinder the development of drugs that utilize these molecules. For instance, HSPs are highly conserved and are expressed widely in both healthy and infected cells, making it difficult to achieve high specificity and selectivity for HSP inhibitors. Consequently, non-specific inhibitors may be toxic and cause damage to normal cells, which limits their therapeutic window (Owen et al., 2016). Additionally, cancer cells and pathogens can develop resistance to HSP inhibitors through various mechanisms, such as the upregulation of compensatory pathways. This resistance poses a challenge to the effectiveness of HSP-targeted therapies. Furthermore, effective delivery and stability are significant limitations for HSP inhibitors in the body due to their poor solubility and bioavailability, ultimately restricting their therapeutic potential. Therefore, there is a need for the development of advanced drug delivery systems. HSPs are part of complex networks that include co-chaperones and client proteins. Disrupting these networks could lead to unforeseen consequences, making it challenging to design effective and safe therapies. Finally, the high costs associated with developing and producing HSP inhibitors can limit their accessibility for low-income patients and countries (Katsogiannou et al., 2014).

## 12 Conclusion and future perspectives

Heat shock proteins are triggered by various adverse cellular conditions, such as harsh environments. These molecules are essential for regulating and preserving proteins within cells. They also help detect unattended or foreign agents that could disturb normal cellular operations, making them valuable biomarkers for atypical cellular activity. Moreover, these proteins are promising options for addressing complex diseases like cancer, malaria, and COVID-19. For example, the proteomics of SARS-CoV-2 depends on the human host’s molecular folding mechanisms. Additionally, *Plasmodium falciparum*, the malaria parasite, depends on heat shock proteins to survive when moving from the mosquito vector to the human body. Cancer cells can exploit heat shock proteins to grow, differentiate, and spread within human cells, positioning these proteins as targets for cancer treatment. In summary, heat shock proteins are integral to both essential and problematic cellular functions. Standardized methods for detecting heat shock proteins are necessary to guarantee consistency and reliability in both research and clinical settings. Sample collection, preparation, and storage are essential components of this process. Consequently, guidelines for handling tissues, protein extraction, and preservation are vital to reduce variability. Implementing reference standards and controls in assays will contribute to accuracy and reproducibility. Additionally, validating detection methods is crucial; this includes evaluating their sensitivity, specificity, and reproducibility. Multi-center validation studies play a key role in ensuring the reliability and applicability of HSP detection methods across various laboratories and populations. Incorporating heat shock protein (HSP) detection techniques into existing diagnostic platforms can significantly improve disease diagnosis efficiency and accuracy. For instance, microfluidic devices, or lab-on-a-chip systems, can combine HSP detection with other diagnostic tests. These systems manage small fluid volumes, facilitating quick and sensitive biomarker detection, including HSPs. Recent research has seen the integration of CRISPR technology into diagnostic platforms, allowing for the rapid and precise detection of nucleic acids and proteins, including HSPs (Owen et al., 2016; Katsogiannou et al., 2014). These systems demonstrate high sensitivity and specificity, which makes them ideal for early disease detection. Finally, conducting a cost-effectiveness analysis for the implementation of heat shock protein detection should consider several aspects: economic evaluation models, direct costs (such as equipment, reagents, and labor), indirect costs (such as patient time and transportation), health outcomes, sensitivity analysis, implementation strategies, and regulatory and reimbursement factors considerations.
